# Temporal Dynamics of the Intestinal Microbiome Following Short-Term Dietary Restriction

**DOI:** 10.3390/nu14142785

**Published:** 2022-07-06

**Authors:** Erik M. Anderson, Jared M. Rozowsky, Brian J. Fazzone, Emilie A. Schmidt, Bruce R. Stevens, Kerri A. O’Malley, Salvatore T. Scali, Scott A. Berceli

**Affiliations:** 1 Department of Surgery, University of Florida College of Medicine, 1600 SW Archer Rd., Gainesville, FL 32610, USA; erik.anderson@surgery.ufl.edu (E.M.A.); jared.rozowsky@surgery.ufl.edu (J.M.R.); brian.fazzone@surgery.ufl.edu (B.J.F.); emilie.schmidt@surgery.ufl.edu (E.A.S.); kerri.omalley@surgery.ufl.edu (K.A.O.); salvatore.scali@surgery.ufl.edu (S.T.S.); 2 Department of Surgery, Malcolm Randall Veteran Affairs Medical Center, 1601 SW Archer Rd., Gainesville, FL 32610, USA; 3 Department of Physiology and Functional Genomics, University of Florida College of Medicine, 1600 SW Archer Rd., Gainesville, FL 32610, USA; stevensb@ufl.edu

**Keywords:** dietary intervention, dietary restriction, caloric restriction, microbiome, intestinal microbiome, pre-operative care

## Abstract

Short-term dietary restriction has been proposed as an intriguing pre-operative conditioning strategy designed to attenuate the surgical stress response and improve outcomes. However, it is unclear how this nutritional intervention influences the microbiome, which is known to modulate the systemic condition. Healthy individuals were recruited to participate in a four-day, 70% protein-restricted, 30% calorie-restricted diet, and stool samples were collected at baseline, after the restricted diet, and after resuming normal food intake. Taxonomy and functional pathway analysis was performed via shotgun metagenomic sequencing, prevalence filtering, and differential abundance analysis. High prevalence species were altered by the dietary intervention but quickly returned to baseline after restarting a regular diet. Composition and functional changes after the restricted diet included the decreased relative abundance of commensal bacteria and a catabolic phenotype. Notable species changes included *Faecalibacterium prausnitzii* and *Roseburia intestinalis*, which are major butyrate producers within the colon and are characteristically decreased in many disease states. The macronutrient components of the diet might have influenced these changes. We conclude that short-term dietary restriction modulates the ecology of the gut microbiome, with this modulation being characterized by a relative dysbiosis.

## 1. Introduction

Dietary restriction, defined as reduced caloric intake without malnutrition, has been proposed as an intriguing pre-operative conditioning strategy to mitigate surgical stress and improve outcomes [1,2,3]. Calorie restriction has been shown to counteract the biological processes of aging via oxidative stress reduction and attenuation of the metabolic and hormonal processes related to disease [4,5,6,7]. However, issues with long-term compliance have limited the implementation of dietary intervention in humans [6,7]. Short-term dietary restriction (stDR) has demonstrated similar benefits, paralleling physiologic patterns of long-term interventions [8,9]. Notably, stDR attenuates insulin resistance, upregulates antioxidant activity, and protects against ischemia-reperfusion injury [9,10,11,12,13]. To date, preclinical surgical models have shown improved stress response patterns and favorable surgical outcomes [14,15,16,17,18]. Furthermore, several pilot studies have shown feasibility and safety in surgical cohorts [19,20,21,22].

Despite encouraging results in clinical and preclinical studies, a clear understanding of the systemic response that results from stDR is lacking. Insights into this question are beginning to emerge. It has been demonstrated that nutrient-sensing pathways (e.g., mTOR, AMPK, insulin/IFG-1, SIRT) are altered after caloric restriction, shifting cellular phenotypes from growth and reproduction to survival and stress resistance [7,8]. Protein restriction, specifically, appears to be the dominant driver of many cell-mediated adaptations [17,23,24,25,26,27,28]. However, differences in macronutrient composition and absorption have produced variable response patterns [29]. The intestinal microbiome, a key regulator of nutrient bioavailability and enteric health, is central to understanding this variability [30,31]. Intestinal physiology affected by the gut microbiome composition further steers whole-body health status [32,33,34,35]. Therefore, dietary interventions targeted to specific compositional and functional patterns within the gut offer the opportunity to control the systemic condition [36,37].

Nutritional restriction likely plays a critical role in the systemic response via the adaptations of the gut microbiome. Animal models suggest that stDR upregulates several commensal species that support favorable homeostatic conditions, such as the preservation of the integrity of the gut membrane, regulatory T cell differentiation, and the production of a favorable cytokine profile. In combination, an overall anti-inflammatory response contributes to the long-term health benefits of a restrictive dietary intervention [6,38,39]. Unfortunately, the bulk of the human microbiome studies evaluating the impact of dietary modulation has been limited to long-term obesity treatment interventions. Particularly, the influence of short-term interventions (less than 1-week), which could be leveraged as a therapeutic opportunity before surgery, has not been explored. As such, community response patterns to stDR remain largely unknown. In the present study, we addressed this knowledge gap by examining the intestinal microbiome’s temporal dynamics during stDR, to better understand the functional mechanisms of the intervention and its implications as a potential pre-operative intervention to improve outcomes.

## 2. Materials and Methods

### 2.1. Study Design

This study was designed to evaluate the dynamic changes in the human intestinal microbiome that result from stDR. The selected diet was a 4-day calorie- and protein-restricted regimen followed by a return to an individual’s typical diet post-intervention. Preliminary findings have demonstrated the safety and feasibility of the diet [19,20]. The study was approved by the Institutional Review Board at the University of Florida.

### 2.2. Subjects and Dietary Intervention

Healthy adult males and females were recruited to participate. Individuals with advanced age (>70 years old) or any significant medical condition (e.g., cancer, diabetes, inflammatory bowel disease, chronic kidney disease) were excluded to maximize the homogeneity among the enrolled participants. All subjects provided informed consent prior to participation. The intervention was a 4-day liquid-based diet, individualized to achieve 30% calorie restriction and 70% protein restriction. A pre-intervention 15% protein caloric intake of 15% was assumed. Each participant’s daily energy requirement was calculated based on resting energy expenditure (REE) plus additional energy needs, estimated using the Paffenbarger physical activity questionnaire [40,41]. The Mifflin St. Jeor equation was used to calculate REE for each subject [42]:

REE = 9.99 × weight (kg) + 6.25 × height (cm) − 4.92 × age (yrs) + 166 × sex (male = 1, female = 0) − 161

Restricted calories-to-be-consumed were based on each subject’s total daily energy requirement. During the 4-day dietary intervention, nutritional intake was limited to the study diet, which was a powder shake (Scandishake^®^ Mix, Nutricia Advanced Medical Nutrition, Utrecht, The Netherlands) mixed with almond milk. Scandishake^®^ Mix is a weight gain supplement selected for its low protein content. Each serving of Scandishake^®^ Mix contains 491 Kcal, which is composed of 4% protein, 42% carbohydrates, and 44% fat [19]. Four shake flavors were available, and water intake was unrestricted. The diet was consumed in an outpatient setting and compliance was monitored with a MealLogger mobile application (www.meallogger.com, accessed on 8 November 2019). Following 4 days of dietary intervention, the participants resumed their baseline dietary habits without restrictions.

### 2.3. Sample Collection, DNA Extraction, and Shotgun Metagenomic Sequencing

Stool samples were collected at three time points: before dietary intervention (“Baseline”), on the last day of dietary restriction (“Day-4”), and three days after resuming an unrestricted diet (“Day-7”). Samples produced on other days of the study period were excluded from analysis. Subjects were provided fecal collection kits, which included a plastic toilet insert, a 1 g-sized scooper, and a DNA/RNA shield fecal collection tube (Zymo Research, Irvine, CA, USA). Samples were delivered to the study team within 24 h of production and tubes were then stored at −20 °C until processing. After thawing, DNA was isolated from each sample using the ZymoBiomics^®^-96 MagBead DNA Kit (Zymo Research, Irvine, CA, USA) followed by shotgun metagenomic sequencing (Zymo Research, Irvine, CA, USA). Whole-genome sequencing libraries were created with the Nextera^®^ DNA Flex Library Prep Kit (Illumina, San Diego, CA, USA) using internal dual-index 8bp barcodes with Nextera^®^ adapters. Quality control and quantification were performed with TapeStation^®^ (Agilent Technologies, Santa Clara, CA, USA), and the libraries were pooled in equal abundance. Final pool was quantified with qPCR and sequenced with NovaSeq^®^ (Illumina, San Diego, CA, USA).

After sequencing, Trimmomatic was used to trim reads and remove adapters and low-quality fractions [43]. A 6bp window size and a cutoff score of 20 were used for sliding window quality filtering. Reads smaller than 70bp were removed. Metagenomic compositional profiling and abundance analysis was then performed with Centrifuge to provide taxonomic information for each sample based on genomic datasets [44]. Functional profiling was performed using HUMAnN2, including the identification of gene families from UniRef protein databases and metabolic pathway identification from the MetaCyc database [45,46,47].

### 2.4. Taxonomic and Functional Pathway Analysis

Species-level α diversity analysis was first performed between the three conditions with ANOVA testing. For unfiltered taxonomy results, measurements of β diversity were quantified at various taxonomic levels (phylum, genus, species) using compositional barplots, permutational multivariate analyses of variance (PERMANOVA) with Bray–Curtis dissimilarity metrics, and abundance heat maps with hierarchical clustering based on Euclidean distances with center-log-ration transformation when appropriate.

To focus the analysis on the prevalent abundant organisms, an established analysis pipeline was utilized to remove noisy spurious data representing species of low prevalence [33,48]. Prevalence filtering was performed using PIME, which uses robust machine learning algorithms at incremental filtering cutoffs to identify an ideal prevalence threshold based on error detection [49]. For filtered, highly prevalent species, exploratory analysis was performed using principal component analysis and measures of conditional variance and β diversity were repeated. Differential abundance analysis was performed using ALDEx2, which has demonstrated its superiority in minimizing variations in collection and processing [50,51]. Sample analysis included multiple instances of Monte Carlo sampling from a Dirichlet distribution, followed by center-log-ratio transformation and Kruskal–Wallis significance testing with false discovery rate (FDR) correction. Pairwise comparisons were then performed based on Welch *t*-testing with correction and effect size calculations to assess differences between conditions.

The evaluation of MetaCyc functional pathways similarly included principal component analysis, hierarchical clustering, and prevalence filtering. Normalized reads were compared between the three conditions using ANOVA testing controlled for FDR. All analyses were performed using R statistical software 4.1.1 and the MicrobiomeAnalyst web-based platform [52]. Network analysis was created using Cytoscape 3.9.1, and a heat tree was created with Metacoder [53,54]. *p* < 0.05 was considered significant.

## 3. Results

### 3.1. Subject Characteristics and Specimen Collection

**Figure 1:**
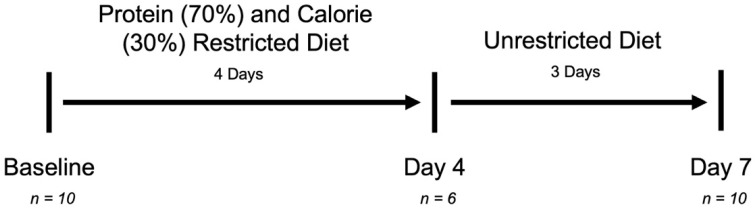
Study Timeline. Ten participants followed a four-day, calorie and protein restriction diet followed by three days of a normal, unrestricted diet. Stool samples were collected at Baseline, on Day-4, and on Day-7. A total of 10 stool samples were available at Baseline and on Day-7. A total of 6 Stool samples were able to be provided on Day-4.

Ten healthy individuals were recruited to participate in the study: five males and five females. The average age was 28.2 (±9.2) years old, with an average BMI of 24.7 (±4.2). In total, 60% of participants were white and 20% used tobacco products. Table 1 shows the height, weight, demographic characteristics, and daily energy requirements for each subject as well as any medication or baseline oral supplement use. Figure 1 depicts the study timeline, including the dietary intervention, timing of sample collections, and number of samples at each time point. Six of the study participants collected stool samples at all three time points. Four individuals did not have a bowel movement on Day-4 of the dietary intervention but were able to provide a baseline sample and a sample after resuming a normal diet for three days.

### 3.2. Compositional Analysis

**Figure 2:**
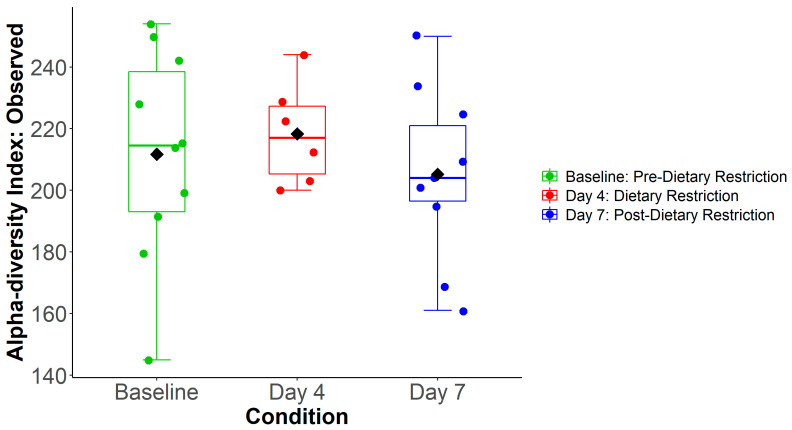
Alpha Diversity. Average number of observed species was not found to be different between conditions.

After quality filtering, a total of 91,410,253 reads were sequenced from 26 samples, with an average of 3,515,779 reads (±95,183.4) per sample. Compositional profiling identified 681 unique bacterial species from 234 genera and 14 phyla. Figure 2 shows the total number of species per sample, classified by condition. Day-4 samples had an average of 218.3 (±16.7) species identified, which was not found to differ from the Baseline (211.7 ± 34.3) or Day-7 (205.2 ± 27.1) conditions (*p* = 0.669). Similarly, there were no differences in other measures of alpha diversity (Shannon index *p* = 0.239, Simpson index *p* = 0.194). PERMANOVA analysis of the unfiltered reads revealed no significant variation at three taxa levels (species *p* = 0.170 species, genus *p* = 0.139, phylum *p* = 0.101). A phylum-level compositional bar plot for each sample is shown in Supplementary Figure S1. Hierarchical clustering revealed grouping by patient, rather than condition, as the dominant factor (Supplementary Figure S2).

**Figure 3:**
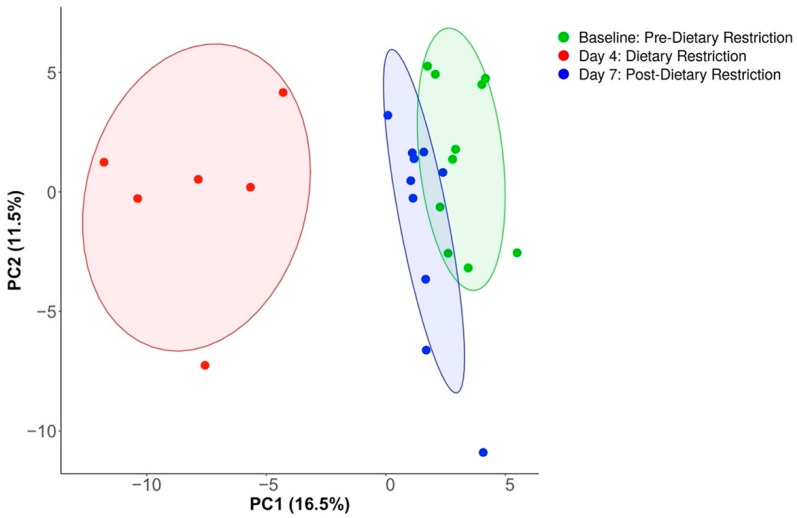
Principal component analysis of species abundance after prevalence filtering. Dietary restriction caused unique species composition, while Baseline and Day-7 conditions showed adjacent clustering, representing group similarities.

**Figure 4:**
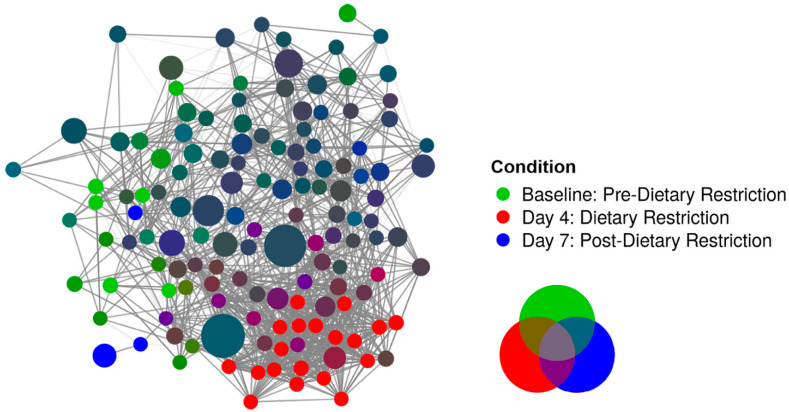
Network analysis of high prevalent species. A total of 136 high prevalent species are depicted by individual nodes, and each node color represents relative abundance by condition. Edges are significant species correlations (*p* < 0.05). Edge intensity depicts the value of the correlation coefficient.

Filtering noisy, spurious species of low prevalence unmasked significant compositional changes associated with restricted dietary intervention. Using a machine learning algorithm, PIME identified 70% prevalence as the ideal cutoff, with an out-of-bin error rate of 0% (Supplementary Table S1). A total of 136 of the 681 species remained at this high prevalence threshold, which corresponded to 74,291,605 of the total 91,410,253 reads (81.3%). Figure 3 shows a principal component analysis of filtered taxa, which demonstrated notable separation as a function of time. Four days of dietary restriction caused distinct species variation among common organisms before returning to baseline, as suggested by adjacent clustering of the Baseline and Day-7 conditions. PERMANOVA confirmed differences between conditions (*p* < 0.001). Figure 4 shows a network analysis of the 136 species with high prevalence. Each species is represented as a node, and conditions are represented as node colors, with various color mixing depending on the relative abundance of a species between conditions. Edges pertain to significant correlations between species (*p* < 0.05). Day-4 again revealed unique clustering, although most of the species unique to that condition had relatively low abundance. Peripheral species in the figure demonstrated an overlap between the Baseline and Day-7 conditions. Supplementary Figure S3 shows condition differences at all analyzed taxonomy levels.

**Figure 5:**
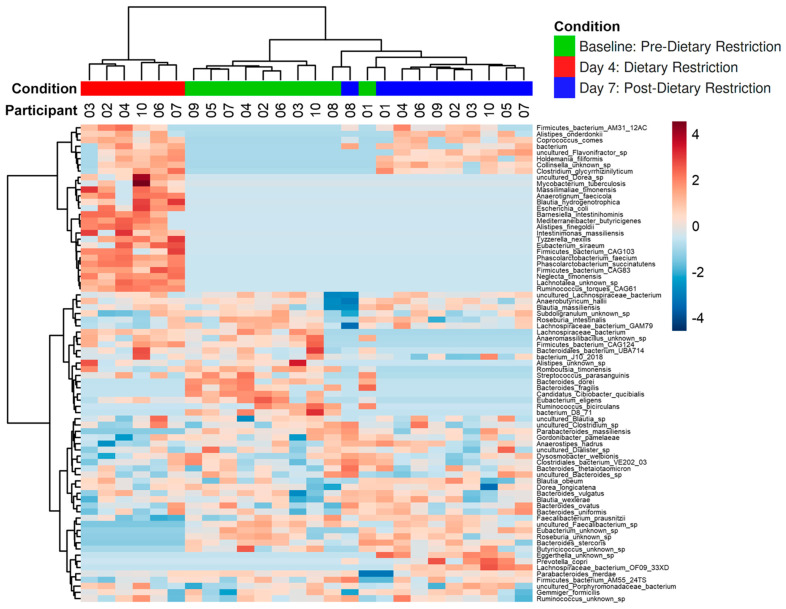
Heatmap of significantly different species (FDR *p* < 0.05). A total of 77 species were found to have differences between conditions (FDR *p* < 0.05). Species grouped well by condition. Most species present at Baseline decreased in abundance on Day-4 and trended toward baseline levels by Day-7. Several species were unique to the Day-4 condition.

ALDEx2 was then used to perform species-level differential abundance analysis to determine which species were driving compositional changes after dietary intervention. Seventy-seven species were found to be significantly different between the three conditions (FDR *p* < 0.05). Figure 5 shows an abundance heat map of significant species with unsupervised clustering. The most prominent species at Baseline demonstrated a marked decrease in abundance after restricted dietary intervention (Day-4). After resuming an unrestricted diet, there was a notable return to the pre-intervention state, with the majority of bacterial species present at Baseline seen in abundance on Day-7. Despite this generalized recovery of the flora, several unique microbial signatures that emerged on Day 4 persisted into Day-7.

**Figure 6:**
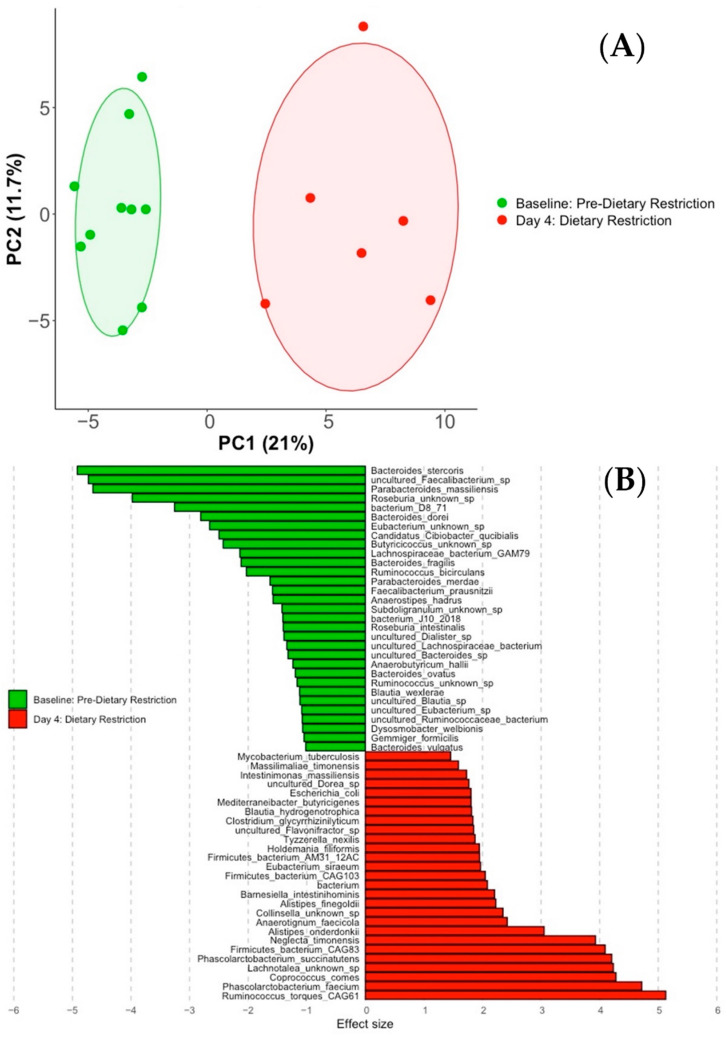
Baseline v. Day-4. Principal component analysis of species present between the two conditions (**A**). Effect size graph of significantly different species (FDR *p* < 0.05, effect size > 1) (**B**).

To further quantify directional changes between conditions, three pairwise comparisons were then performed: Baseline v. Day-4, Day-4 v. Day-7, and Baseline v. Day-7. Figure 6A shows a principal component analysis of Baseline and Day-4 species present at the 70% prevalence threshold (*n* = 133). Distinct differences between the two conditions are obvious. ALDEx2 differential abundance analysis identified 63 species with significant variation after dietary intervention (*p* < 0.05). Supplementary Table S2 lists these 63 species along with their higher taxa levels and effect sizes. Species with an effect size greater than 1 are shown in Figure 6B, which indicates variations between group greater than within group dispersion. Negative effect size values favor the baseline condition, while positive values favor the Day-4 condition. Dietary restriction caused a decreased relative abundance of several commensal bacteria, including *Faecalibacterium prausnitzii* (FDR *p* = 0.004, effect size = −1.58), *Roseburia intestinalis* (FDR *p* = 0.010, effect size = −1.40), *Anaerostipes hadrus* (FDR *p* = 0.003, effect size = −1.57), *Anaerobutyricum hallii* (FDR *p* = 0.025, effect size = −1.24), *Blautia wexlerae* (FDR *p* = 0.016, effect size = −1.12), *Ruminococcus bicirculans* (FDR *p* = 0.002, effect size = −2.03), and *Dysosmobacter welbionis* (FDR *p* = 0.013, effect size = −1.07). Also, the following *Bacteroides* species universally decreased after the dietary intervention: *Bacteroides stercoris* (FDR *p* < 0.001, effect size = −4.92), *Bacteroides dorei* (FDR *p* < 0.001, effect size = −2.81), *Bacteroides fragilis* (FDR *p* = 0.002, effect size = −2.12), *Bacteroides ovatus* (FDR *p* = 0.028, effect size = −1.2), and *Bacteroides vulgatus* (FDR *p* = 0.017, effect size = −1.02). Well studied species that increased on Day-4 included *Ruminococcus torques* (FDR *p* < 0.001, effect size = 5.13), *Coprococcus comes* (FDR *p* < 0.001, effect size = 4.28), *Escherichia coli* (FDR *p* = 0.030, effect size = 1.81), *Mycobacterium tuberculosis* (FDR *p* = 0.043, effect size = 1.46), and *Alistipes finegoldii* (FDR *p* = 0.026, effect size = 2.23).

Supplementary Figure S4 and Supplementary Table S3 show similar analyses of the species present on Day-4 and Day-7. Sixty-four species significantly varied between the two conditions (*p* < 0.05). Interestingly, most species reverted to their original composition after resuming an unrestricted diet. For example, *Faecalibacterium prausnitzii* (FDR *p* = 0.001, effect size = 2.21), *Anaerostipes hadrus* (FDR *p* = 0.001, effect size = 1.68), *Anaerobutyricum hallii* (FDR *p* = 0.020, effect size = 1.12), *Blautia wexlerae* (FDR *p* = 0.0004, effect size = 1.53), and *Dysosmobacter welbionis* (FDR *p* = 0.011, effect size = 1.17) had reciprocal changes in effect sizes compared to Baseline v. Day-4. Similarly, *Bacteroides* species uniformly increased on Day-7. Species that were increased on Day-4 were also downregulated by Day-7, including *Ruminococcus torques* (FDR *p* < 0.001, effect size = −5.13), *Escherichia coli* (FDR *p* = 0.029, effect size = −1.88), and *Alistipes finegoldii* (FDR *p* = 0.032, effect size = −2.36). Supplementary Figure S5 and Supplementary Table S4 depict Baseline v Day-7 analyses to further support the similarity between the two conditions. Principal component analysis revealed group overlap, and 24 species differed between Baseline and Day-7 (*p* < 0.05), which is much fewer than the two previous comparisons. *Romboutsia timonensis* (FDR *p* < 0.001, effect size −3.92), *Eubacterium eligens* (FDR *p* = 0.004, effect size −1.91), *Streptococcus parasanguinis* (FDR *p* = 0.005, effect size −1.91), *Prevotella copri* (FDR *p* = 0.004, effect size 1.61), and *Holdemania filiformis* (FDR *p* = 0.002, effect size 2.06) are a few notable bacteria that were different between baseline and Day-7.

### 3.3. Functional Pathway Analysis

**Figure 7:**
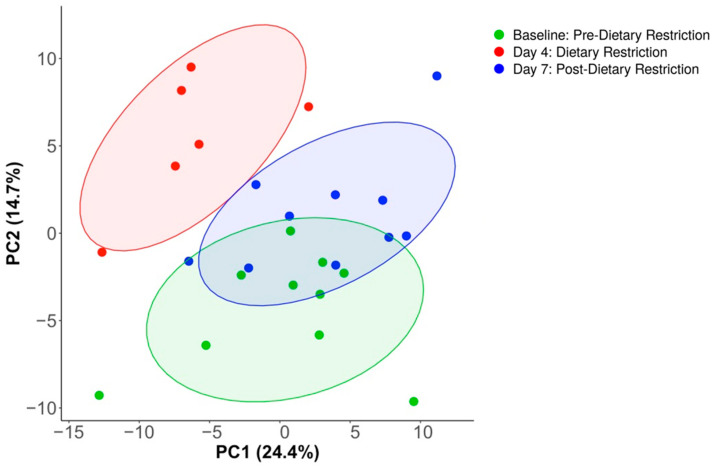
Principal component analysis of MetaCyc pathway abundance after prevalence filtering. Similar to the findings of taxonomy analysis, dietary restriction created unique clustering on Day-4, while Baseline and Day-7 conditions showed overlap clustering, representing group similarities.

Functional pathway analysis identified 345 unique MetaCyc pathways. Using PIME, a prevalence cutoff of 85% was identified, which included 177 pathways. Similar to the findings of compositional analysis, conditional variation was present after prevalence filtering, including adjacent positioning of Baseline and Day-7 conditions (Figure 7). A total of 45 of the 177 pathways had significant differences between the three conditions (FDR *p* < 0.05). All 45 were identified as either biosynthesis, degradation, or precursor metabolite pathways based on MetaCyc ontology classification.

**Figure 8:**
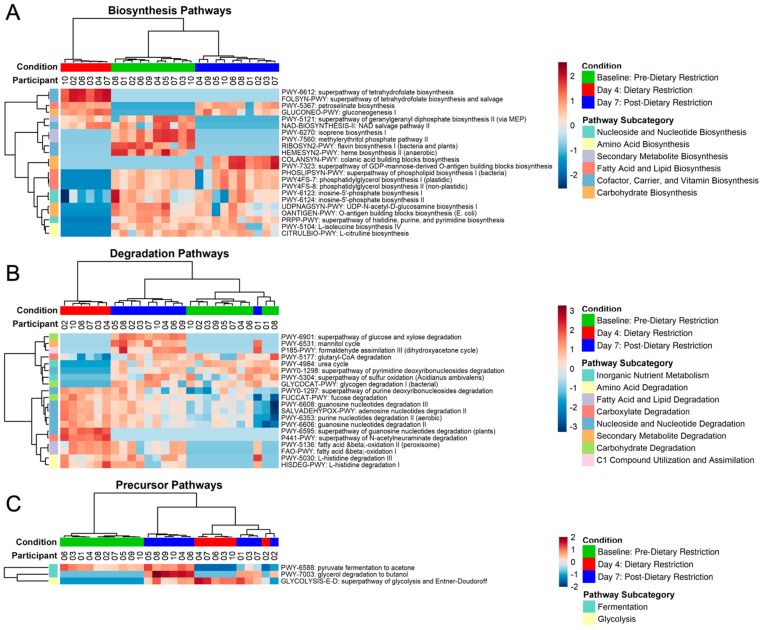
Heatmap of significantly different pathways (FDR *p* < 0.05). Based on MetaCyc ontology classification, pathways were categorized as biosynthesis (**A**), degradation (**B**), or precursor metabolic pathways (**C**).

Figure 8 shows the hierarchical clustering of the three pathway types. Conditions and pathway subcategories both group together. The subcategories describe the biological function of the individual pathways, which is based on the metabolites produced or consumed by the pathway of interest. Many biosynthesis pathways decreased after dietary restriction, including fatty acid and lipid biosynthesis, nucleoside and nucleotide biosynthesis, and carbohydrate biosynthesis (Figure 8A). Most of these metabolic pathways increased back to near-Baseline levels after resuming an unrestricted diet. Degradation pathways are shown in Figure 8B, which, alternatively, were mostly upregulated on Day-4, particularly the amino acid, fatty acid, and nucleoside/nucleotide degradation pathways. Most of these active degradation pathways persisted on Day-7. Finally, fermentation pathways were downregulated after stDR (Figure 8C).

## 4. Discussion

Brief (<1 week) dietary restriction has been proposed as a novel pre-operative conditioning strategy, designed to attenuate the surgical stress response and improve postoperative outcomes. Clinical trials investigating its safety, feasibility, and preliminary outcomes have already been performed in humans based on reassuring preclinical data. However, the systemic influence of dietary intervention is not fully understood; particularly, it is unknown how short-term protein and calorie restriction influences resident microbiota. In this study, we demonstrated that stDR causes distinct, rapid, and reversible alterations in the composition of the intestinal microbiome that correspond to changes in its functional phenotype. Interestingly, a relative dysbiosis was noted after dietary intervention. These microbial transformations have implications related to the systemic condition, especially as a pre-operative intervention.

Initial evaluation of the unfiltered taxa revealed no differences in measures of α and β diversity between the three conditions. Additionally, hierarchical clustering analysis revealed grouping by participant rather than the intervention, indicating that each individual’s microbiota mostly remained uniquely characterized throughout the study. This was not unexpected and was seen in the work of Salonen et al., who showed that inter-individual variance predominates compositional analysis after dietary interventions, with three different diets explaining only about 10% of the total variance in their study [55]. Additionally, Wu et al. showed that individuals have underlying microbial signatures that correlate with long-term dietary habits but are maintained despite acute changes in food intake [56]. In fact, 70% of species identified from obese patients undergoing fat or carbohydrate restriction were unique to each individual, which matches our taxonomy prevalence filtering cutoff [57]. After prevalence filtering, we were able to eliminate these individual ecology patterns and identify distinct community variations secondary to the dietary intervention.

We found protein and calorie restriction to cause a decrease in several short chain fatty acid (SCFA)-producing commensal bacteria. *Faecalibacterium prausnitzii* and *Roseburia intestinalis* are particularly notable species that were influenced by the diet. Both species are consistently reported as the primary butyrate producers of the intestine [58,59]. Butyrate is the most bioactive SCFA, functioning as the primary energy source for colonocytes and thereby promoting colonic health and barrier integrity. In addition, butyrate has been shown to have anti-inflammatory properties, including NF-kB pathway suppression and regulatory T cell differentiation [32,33,60]. SCFAs are produced via the bacterial fermentation of dietary fibers and resistant starch, so fiber supplementation is thought to be protective by providing substrates to support resident flora and increasing products which stabilize enteric homeostasis [61]. For their beneficial metabolic profile, many of these commensal organisms are probiotic candidates [62]. Therefore, a decreased relative abundance of these species disrupts their health benefits, suggesting a disadvantageous effect of the diet.

Butyrate-producing commensal species are known to have a decreased relative abundance in many disease states. Colonic diseases, such as inflammatory bowel disease, irritable bowel syndrome, and colorectal cancer, have been shown to be related to microbial dysbiosis [63]. Interestingly, systemic metabolic diseases, such as obesity, diabetes, as well as cardiovascular disease, have similar imbalance characteristics, linking the microbiome to both local and systemic health [64,65]. For example, *Blautia wexlerae* were depleted in obese patients, with it having been related to intestinal inflammation [66,67]. Additionally, *Dysosmobacter welbionis* negatively correlated with BMI, fasting glucose, and glycated hemoglobin, and oral treatment has been shown to counteract insulin resistance and inflammation [68]. *Faecalibacterium prausnitzii* or *Roseburia intestinalis* has been found to be decreased in all of the aforementioned medical conditions [58,59,63,69].

Several *Bacteroides* species were also decreased after dietary restriction. *Bacteroides stercoris*, *Bacteroides dorei*, *Bacteroides fragilis*, *Bacteroides ovatus*, and *Bacteroides vulgatus* are all considered members of the *Bacteroides fragilis* group for their phylogenic relationships and similar functional profiles [70]. *Bacteroides* species are commonly isolated anaerobes from gastrointestinal, abdominal, and perianal infections due to their virulence profile and antibiotic resistance patterns. However, despite their commonly described infection patterns, they are actually commensal bacteria without pathogenic characteristics in a healthy environment [70]. *Bacteroides* is the most abundant genus in the intestine, and supplementation can strengthen the gut barrier and protect against LPS translocation [71]. *Bacteroides*, however, have variable responses to several disease states. *Bacteroides vulgatus* and *Bacterdoies dorei* have been associated with inflammatory bowel disease, irritable bowel disease, celiac disease, and other autoimmune conditions [70,72]. However, the same species have been found to be decreased in patients with obesity, type 2 diabetes, and atherosclerotic disease, suggesting protective effects [71,73,74]. Also, *Bacteroides ovatus* is being investigated as a possible probiotic for its anti-inflammatory potential [75].

Despite the downregulation of many commensal bacteria after stDR, the Day-4 condition maintained its species-level α diversity. Network analysis revealed that many of the species upregulated after dietary intervention had low relative abundance but were unique with respect to the Day-4 condition, which explains why the total number of species was unchanged even though baseline communities decreased. *Ruminococcus torques*, which has been shown to have mucin degrading properties and is associated with decreased gut barrier integrity, had the greatest increase in relative abundance after stDR [76,77]. Fecal isolations of individuals with Crohns disease and irritable bowel syndrome have shown increases in *Ruminococcus torques* [78,79]. In addition, several *Alistipes* species which have been linked to cardiovascular disease and colon cancer increased after stDR [80]. However, mice treated with *Alistipes finegoldii* were found to have attenuated colitis, so the significance of the results is unclear [81]. Finally, an increased abundance of *Escherichia coli* favors microbial imbalance after stDR due to its well-described pathogenicity [82].

Pathway analysis was suggestive of a catabolic state after dietary restriction. Macronutrient biosynthesis pathways were mostly reduced on Day-4, although the nicotinamide adenine dinucleotide (NAD) salvage pathways and gluconeogenesis were notable exceptions, with both having previously been found to be elevated in calorie-restricted conditions [83,84]. NAD salvage is required for Sir2 histone deacetylase activity, which can extend the lifespan via DNA silencing during caloric restriction [83,84]. Also, under calorie-restricted conditions, a shift from fermentation to respiration occurs due to the increased efficiency of ATP production, with an excess of ATP being associated with gluconeogenesis [84]. Decreases in two fermentation pathways after stDR support this adaptive change in energy production. Matching the overall reduction in biosynthesis, several degradation pathways were increased on Day-4. Amino acids, fatty acids, and nucleosides/nucleotides are required for energy utilization during limited intake. Obese patients undergoing very low-calorie restriction (800 kcal per day) for 8 weeks also showed a functional phenotype shift, with their catabolic processes being increased in favor of energy utilization from available nutrients [85]. Pathway analysis from inflammatory bowel disease patients has also shown downregulated metabolic processes, particularly amino acid and short chain fatty acid production, suggesting that disease states and nutrient-limited conditions may have overlapping functional impairments and limited systemic bioavailability [86].

Interestingly, the stDR stimulated these adaptations rapidly, in as few as four days. Also, the microbiome quickly reversed back to its baseline condition within 3 days of resuming a regular, unrestricted diet. This return to baseline occurred regardless of each individual’s baseline diet composition. In a similar fashion, pathway analysis revealed an overlap before and after dietary intervention, although several degradation pathways were elevated on Day-7. Therefore, resuming a nutrient-dense diet allows for biosynthetic processes to resume while breakdown processes continue to function, with a slower return to baseline. Considering dietary restriction as a pre-operative conditioning strategy, it is essential to know that rapid, reproducible changes are possible in a short period of time in order to maintain patient compliance. Similar to our results, David et al. also showed that β diversity metrics could be altered after four days of an animal-based diet and return to baseline within five days of stopping the diet [87]. Also, Wu et al. found that composition changes are seen even as early as the first day of dietary intervention [56].

Overall, species-level variation and pathway analysis revealed a state of dysbiosis and stress within the gastrointestinal system after stDR. These community alterations contrast with previously reported microbial adaptations after calorie restriction. Most studies investigating the influences of dietary restriction on the microbiome were murine models utilizing 25–70% restriction with a longer duration, ranging from several weeks to lifelong interventions [88,89,90,91,92]. Rinninella et al. summarize the metabolic implications of the dietary interventions and individual species implicated in these adaptations [38]. The overall benefits are due to improved mucosal barrier integrity, decreased fat mass, improved glucose homeostasis and lipid profiles, and attenuated inflammatory pathways. Wang et al. demonstrated that mice with antibiotic-depleted microbiota were resistant to metabolic changes as a result of caloric restriction, which causally links intestinal community changes to systemic adaptations [56]. *Lactobacillus*, *Bifidobacteria*, and *Bacteroides* species are most commonly increased after restriction diets. *Lactobacillus* is a known probiotic and has been shown to treat obesity by decreasing free fatty acid absorption [93,94]. Furthermore, mice with caloric restriction supplemented with *Lactobacillus* had increased antioxidant activity [95]. *Lactobacillus* inhibits pathogen adhesions and maintains gut barrier integrity [88]. *Bifidobacteria* follow the same ecological patterns and has similar beneficial influences [96]. However, after stDR, we did not find abundance changes for any *Lactobacillus* or *Bifidobacteria* species, and *Bacteroides* species decreased in abundance rather than increased. Due to noticeable community differences, conclusions from these preclinical trials cannot be extrapolated to our dietary intervention.

The influence of caloric restriction on the human microbiome has primarily been investigated in obesity treatment studies. Interventions usually involve very low-calorie restriction (e.g., 800 Kcal/day) with a duration of at least several weeks [85,97,98]. Von Schwartzenberg et al. recently reported that this extreme caloric restriction disrupted the resident microbiota and promoted pathogenic colonization [85]. These conclusions could provide insight into our Day-4 species variation; it is possible that alpha diversity was maintained after stDR due to the loss of colonization resistance and upregulation of non-commensal bacteria. However, most of the microbiome characteristics in these obesity studies do not match our findings, which is understandable due to the differences in dietary intervention and patient population.

The nutritional composition of our study diet likely explains most of the species-level variation that we found after stDR. Possible detrimental elements of the Scandishake^®^ Mix diet include its high fat and carbohydrate content and lack of fiber. Excessive fat and carbohydrates lead to metabolic syndromes with intestinal communities similar to those found in the Day-4 microbiome, such as low abundances of *Faecalibacterium prausnitzii*, *Roseburia intestinalis*, and *Bacteroides* species [73,74]. Zhang et al. found that lifelong caloric restriction in mice produced different microbial signatures depending on the fat content of the diet, and high proportions of fat failed to promote a longevity benefit [99]. This was attributed to higher endotoxin production and increased gut permeability, resulting in systemic inflammation and disease. Detrimental effects can also be seen on a short timescale. Carmody et al. demonstrated that a high fat and high carbohydrate diet rapidly induced dysbiosis, taking 3.5 days to reach a steady state [100]. That timeline matches the four days of dietary restriction in this study. In the context of a protein-restricted diet, Wali et al. demonstrated that even the type of carbohydrate content influences systemic response patterns [101]. High carbohydrate diets that are dense in resistant starches produce the healthiest metabolic outcomes, likely due to the promotion of SCFA-producing bacteria. Conversely, simple sugars, the primary carbohydrates in the Scandishake^®^ Mix, were associated with worse health outcomes. Solon-biet et al. recently demonstrated that the ratio of macronutrients dictated metabolic health and longevity more than restriction, emphasizing the health benefits of a low protein ratio in an *ad libitum* diet [102,103]. In humans, a moderate carbohydrate, moderate protein diet had favorable measures of metabolic health compared to a high carbohydrate, low protein diet [104]. Therefore, calorie restriction and protein restriction may not have additive health benefits and could be detrimental when combined in excess. This would explain the intestinal dysbiosis created by our study diet.

Preclinical and clinical restriction studies are highly variable in their design, utilizing different durations, degrees of restriction, and nutrient compositions, which makes leveraging their results difficult when constructing a pre-operative dietary intervention. Calorie-only restriction has been tested preoperatively with mixed results. Grundmann et al. utilized a 7-day, 40% calorie-restricted diet before cardiac surgery, which was protective against acute kidney injury [22]. However, 3 days of 30% caloric restriction did not improve kidney function outcomes in living donor kidneys [21]. Jongbloed et al. first designed the Scandishake^®^ Mix diet that we used in this study and tested its feasibility in healthy kidney donor and bariatric surgery patients [20]. A total of 20 of the 28 patients (71%) completed the diet, reporting only mild discomforts of hunger and nausea. Preliminary results revealed decreases in the serum levels of branched chain amino acids, which can have health benefits [105]. Therefore, the diet may have a systemic immunomodulatory role despite our findings of a microbial imbalance. Kip et al. subsequently tested the liquid diet on eight vascular surgery patients, with 100% compliance [19]. Pre-operative improvements in insulin sensitivity were noted, with no differences in surgical outcomes compared to the control group. There were no differences in circulating cytokines or adipokines, although it was a limited sample size. The metabolic and immunologic response patterns of the Scandishake^®^ Mix diet are otherwise unknown. Further investigation is needed to determine the systemic influences of the intestinal microbiome after stDR, especially since these factors may influence surgical outcomes.

While healthy subjects were ideal candidates for this preliminary investigation, this cohort was also a study limitation. Age and comorbidities are known to influence the microbiome, so it is possible that the disruptive effects of stDR are blunted or augmented in communities that have a baseline state of dysbiosis. Unfortunately, our results cannot be extrapolated to other populations. Also, surgical interventions were not performed in this study after dietary restriction, so the effects of operative stress on the altered microbiome remain unknown. Other limitations were the small sample size and lack of a non-calorie-restricted control group consuming the Scandishake^®^ Mix diet.

## 5. Conclusions

In conclusion, individual community characteristics of the gut microbiome were preserved after short-term protein and calorie restriction, although distinct changes were noted for species with high prevalence. Surprisingly, intestinal dysbiosis occured after a short-term, high fat, high carbohydrate, low-protein diet, characterized by decreased commensal bacteria and a phenotype shift towards catabolism. These changes occurred rapidly and reversed equally as fast. It is unclear how these community characteristics influence the systemic condition and modulate the surgical stress response. Nevertheless, the results of the present study demonstrate that the microbiome is influenced by a short-term pre-operative diet, which sets the stage for future interventional studies.

## Figures and Tables

**Table 1:** Participant characteristics.

Participant	Gender	Race/ Ethnicity	Age	Height (cm)	Weight (kg)	BMI	Tobacco Use	Medications/ Supplements	Daily Energy Requirement (kcal)
1	F	Asian	22	160	60	23.4	No	Oral contraceptive	1940
2	M	White	30	188	102	28.9	No	-	3267
3	M	White	26	191	98	26.9	No	Magnesium, krill oil	2983
4	M	Pacific Islander	51	188	88	24.9	Yes	-	3078
5	F	White	21	185	70	20.5	No	-	3177
6	M	White	28	188	79	22.4	No	-	2884
7	F	Hispanic	23	168	91	32.2	No	Oral contraceptive	2116
8	F	Asian	33	152	50	21.6	No	-	1738
9	F	White	19	175	58	18.9	No	Oral contraceptive	2221
10	M	White	29	178	86	27.1	Yes	-	3118

## Supplementary Materials

The following are available online at https://www.mdpi.com/article/10.3390/nu14142785/s1, Figure S1: Phylum-level diversity; Figure S2: Hierarchical clustering of unfiltered species; Figure S3: Heat tree comparing condition variation at all taxonomy levels; Figure S4: Day-4 v. Day-7. Principal component analysis of species present between the two conditions (A). Effect size graph of significantly different species (FDR *p* < 0.05, effect size > 1) (B); Figure S5: Baseline v. Day-7. Principal component analysis of species present between the two conditions (A). Effect size graph of significantly different species (FDR *p* < 0.05, effect size > 1) (B); Table S1: Species PIME prevalence filtering output; Table S2: Significant different species between Baseline and Day-4 conditions; Table S3: Significantly different species between Day-4 and Day-7 conditions; Table S4: Significantly different species between Baseline and Day-7 conditions.
