# Supplementary material for: Temporal Dynamics of the Intestinal Microbiome Following Short-Term Dietary Restriction
Source: Nutrients. 2022 Jul 6;14(14):2785. doi: 10.3390/nu14142785 (PMC9318361; doi:10.3390/nu14142785)
Supplement: Supplementary file 1 [file nutrients-14-02785-s001.zip › nutrients-1791459-supplementary.pdf]

nutrients

MDPI

Supplemental Figures/Tables

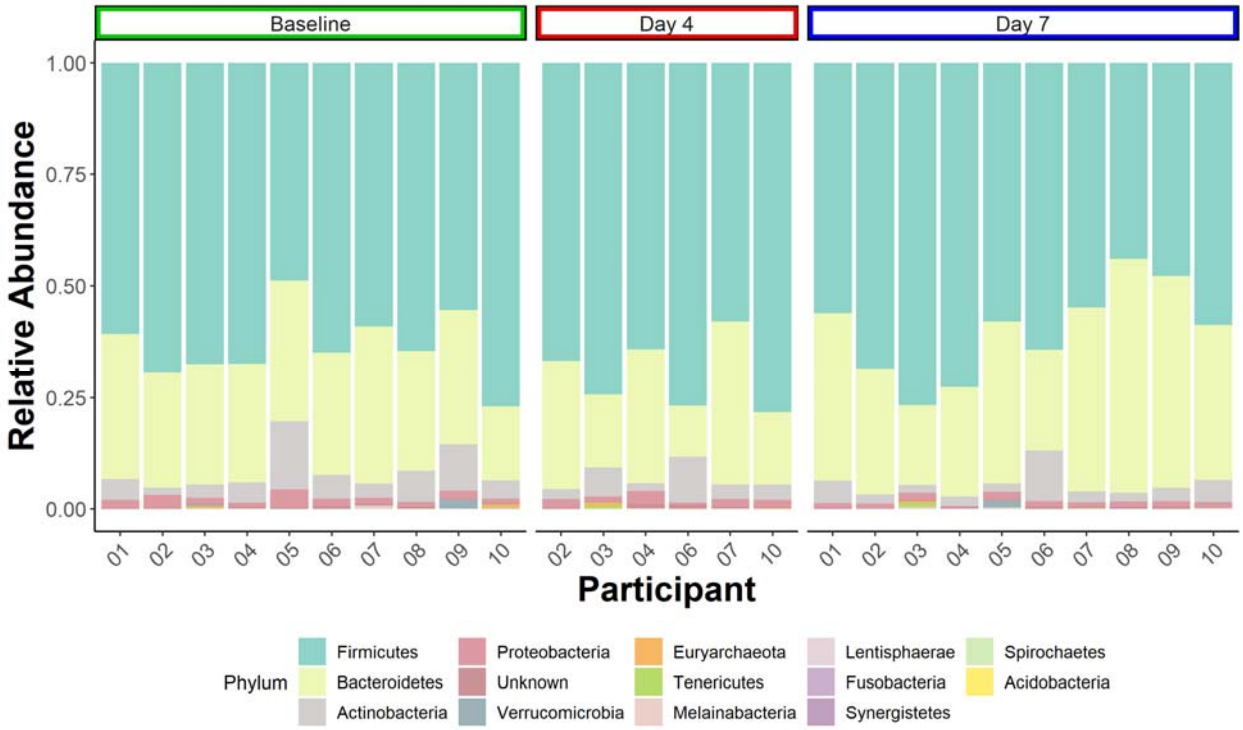

Supplemental Figure S1. Phylum-level Diversity. The relative abundance of each phylum is depicted for each participant at each time-point.

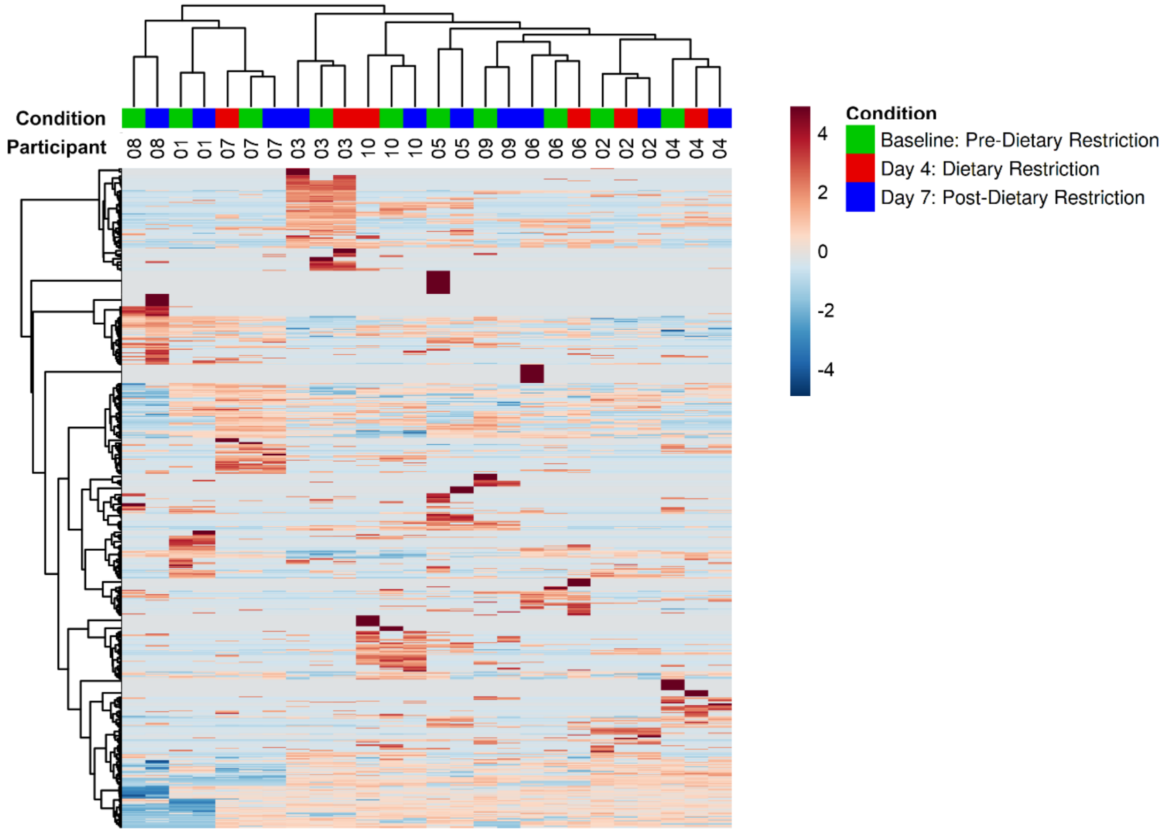

Supplemental Figure S2. Hierarchical clustering of unfiltered species. Species abundance patterns group by patient rather than condition.

Supplemental Table S1. Species PIME prevalence filtering output. Random forests were built at sequential prevalence intervals to determine the ideal cut off value. Out-of-bin (OOB) error rate of 0% was achieved at a 70% prevalence cut off. 74,291,605 reads corresponding to 136 species were present above this filtering threshold.

Prevalence Interval (%)	OOB Error Rate (%)	OTUs (N)	Sequences (N)
5	96.15	681	91,410,253
10	92.31	530	90,013,248
15	96.15	530	90,013248
20	92.31	354	88,450,002
25	84.62	354	88,450,002
30	80.77	313	86,738,175
35	53.85	268	86,252,647
40	30.77	241	83,549,022
45	46.15	241	83,549,022
50	7.69	191	80,761,545
55	3.85	191	80,761,545
60	3.85	176	78,332,889
65	7.69	176	78,332,889
70	0	136	74,291,605
75	3.85	136	74,291,605
80	0	128	68,893,931
85	0	104	67,269,799
90	0	92	59,055,438
95	0	92	59,055,438

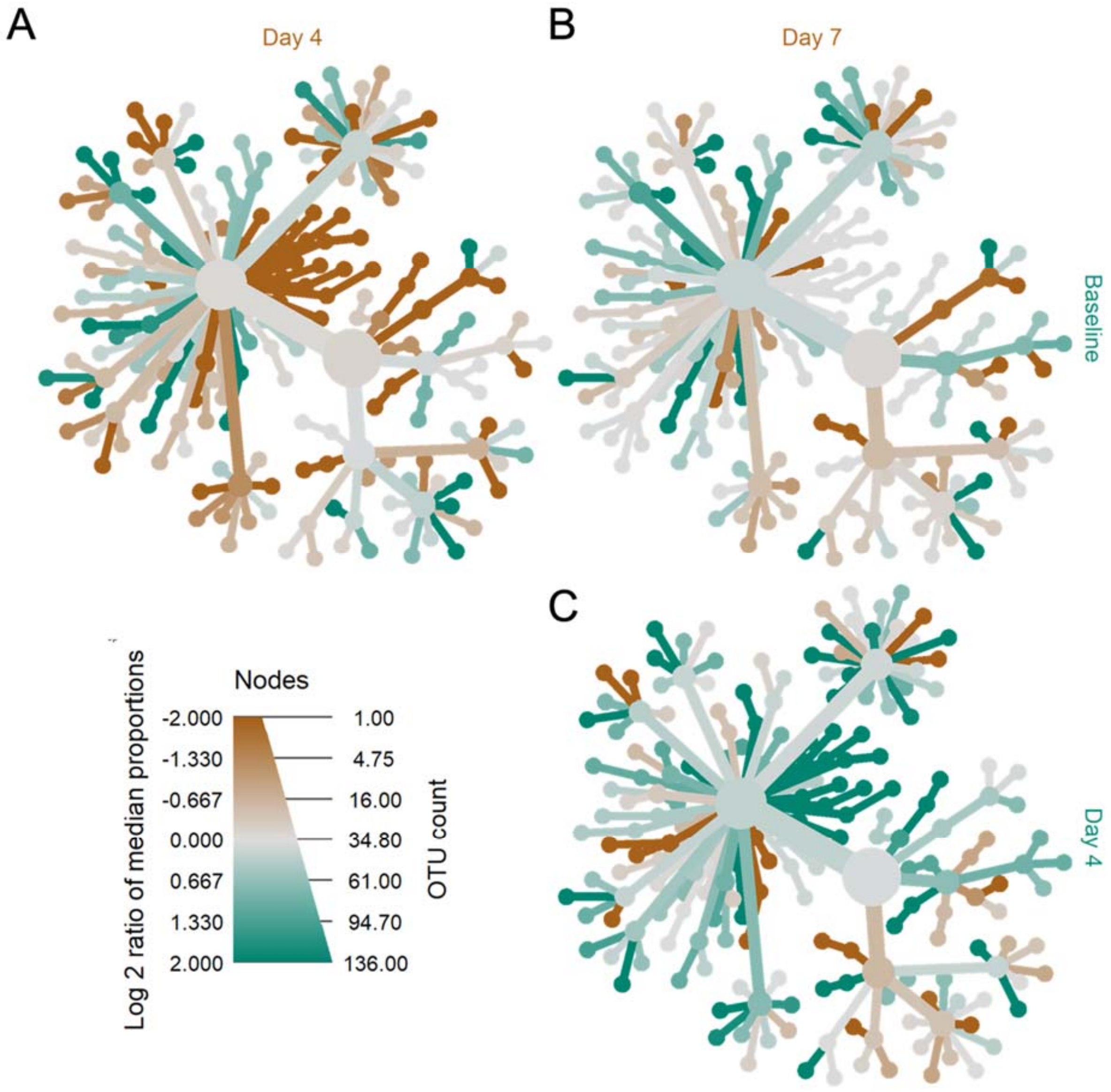
A
Day 4
B
Day 7
Baseline
C
Day 4
Nodes
Log 2 ratio of median proportions
-2.000
-1.330
-0.667
0.000
0.667
1.330
2.000
OTU count
1.00
4.75
16.00
34.80
61.00
94.70
136.00

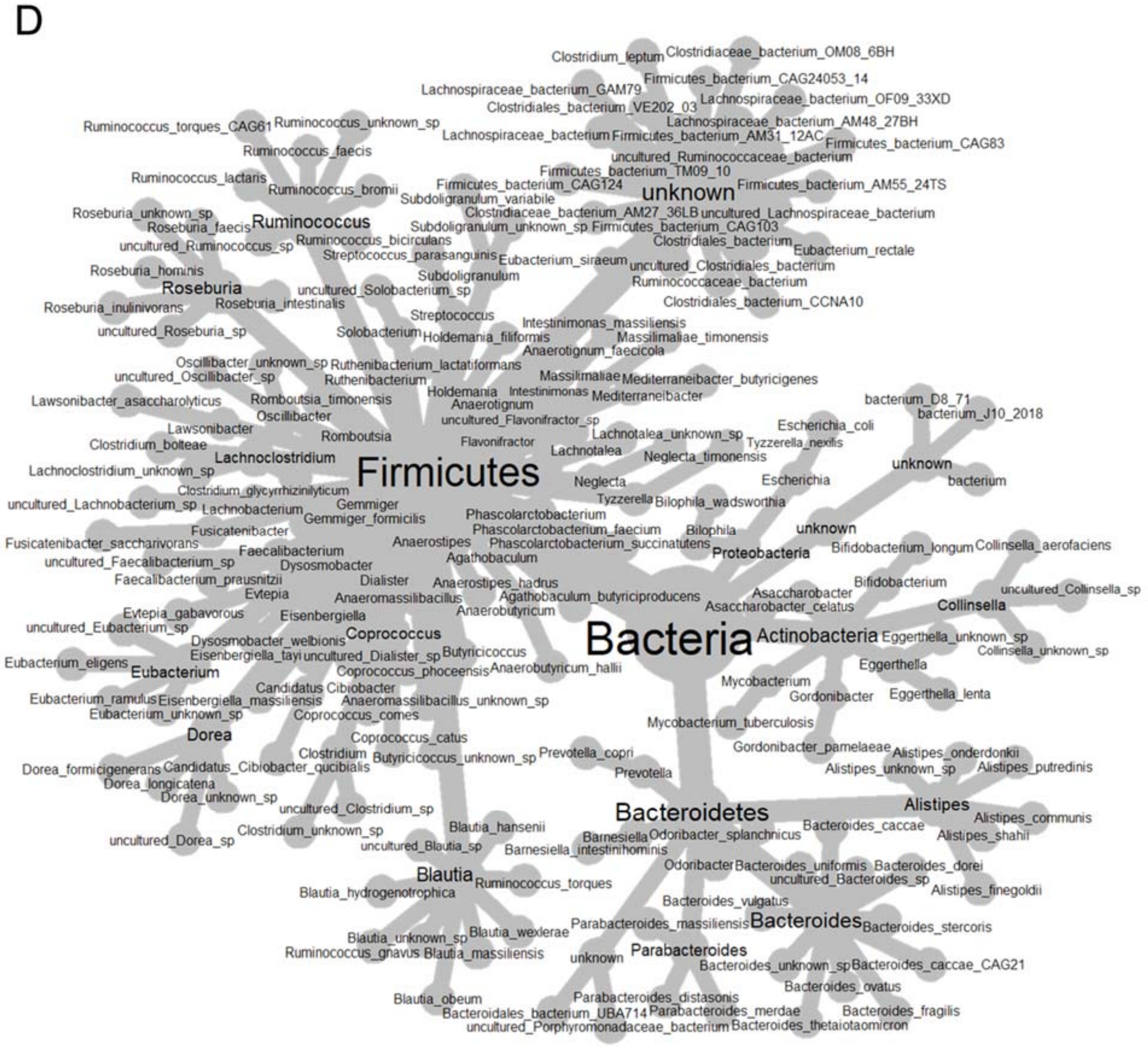

Supplemental Figure S3. Heat tree comparing condition variation at all taxonomy levels. Pairwise comparisons between the three conditions (**A-C**). Size of each node represents the number of species contained within that group. The color of each node shows the favored condition. Comparisons were performed using Wilcox rank-sum test for median read proportion of paired conditions. Heat tree taxa legend (**D**). Kingdom, phylum, genus, and species taxonomy levels were included.

Supplemental Table S2. Significantly different species between Baseline and Day-4 conditions (FDR p<0.05).

Kingdom	Phylum	Genus	Species	Effect Size	FDR p-value
Bacteria	Bacteroidetes	Bacteroides	Bacteroides_stercoris	-4.92	<0.001
Bacteria	Firmicutes	Faecalibacterium	uncultured_Faecalibacterium_sp	-4.73	<0.001
Bacteria	Bacteroidetes	Parabacteroides	Parabacteroides_massiliensis	-4.65	<0.001
Bacteria	Firmicutes	Roseburia	Roseburia_unknown_sp	-3.98	<0.001
Bacteria	unknown	unknown	bacterium_D8_71	-3.26	<0.001
Bacteria	Bacteroidetes	Bacteroides	Bacteroides_dorei	-2.81	<0.001
Bacteria	Firmicutes	Eubacterium	Eubacterium_unknown_sp	-2.66	<0.001
Bacteria	Firmicutes	Candidatus Cibiobacter	Candidatus_Cibiobacter_qucibialis	-2.50	0.002
Bacteria	Firmicutes	Butyricicoccus	Butyricicoccus_unknown_sp	-2.43	0.002
Bacteria	Firmicutes	unknown	Lachnospiraceae_bacterium_GAM79	-2.14	0.001
Bacteria	Bacteroidetes	Bacteroides	Bacteroides_fragilis	-2.12	0.002
Bacteria	Firmicutes	Ruminococcus	Ruminococcus_bicirculans	-2.03	0.002
Bacteria	Firmicutes	Faecalibacterium	Faecalibacterium_prausnitzii	-1.58	0.004
Bacteria	Firmicutes	Anaerostipes	Anaerostipes_hadrus	-1.57	0.003
Bacteria	Firmicutes	Subdoligranulum	Subdoligranulum_unknown_sp	-1.42	0.037
Bacteria	unknown	unknown	bacterium_J10_2018	-1.41	0.012
Bacteria	Firmicutes	Roseburia	Roseburia_intestinalis	-1.40	0.010
Bacteria	Firmicutes	Dialister	uncultured_Dialister_sp	-1.39	0.005
Bacteria	Firmicutes	unknown	uncultured_Lachnospiraceae_bacterium	-1.34	0.005
Bacteria	Bacteroidetes	Bacteroides	uncultured_Bacteroides_sp	-1.32	0.011
Bacteria	Firmicutes	Anaerobutyricum	Anaerobutyricum_hallii	-1.24	0.025
Bacteria	Bacteroidetes	Bacteroides	Bacteroides_ovatus	-1.20	0.028
Bacteria	Firmicutes	Ruminococcus	Ruminococcus_unknown_sp	-1.17	0.015
Bacteria	Firmicutes	Blautia	Blautia_wexlerae	-1.12	0.016
Bacteria	Firmicutes	Blautia	uncultured_Blautia_sp	-1.12	0.025
Bacteria	Firmicutes	Eubacterium	uncultured_Eubacterium_sp	-1.09	0.040
Bacteria	Firmicutes	unknown	uncultured_Ruminococcaceae_bacterium	-1.08	0.034
Bacteria	Firmicutes	Dysosmobacter	Dysosmobacter_welbionis	-1.07	0.013
Bacteria	Firmicutes	Gemmiger	Gemmiger_formicilis	-1.05	0.024
Bacteria	Bacteroidetes	Bacteroides	Bacteroides_vulgatus	-1.02	0.017
Bacteria	Bacteroidetes	Bacteroides	Bacteroides_thetaiotaomicron	-1.00	0.030
Bacteria	Firmicutes	Clostridium	uncultured_Clostridium_sp	-0.96	0.031
Bacteria	Firmicutes	unknown	Clostridiales_bacterium_VE202_03	-0.96	0.015
Bacteria	Firmicutes	Fusicatenibacter	Fusicatenibacter_saccharivorans	-0.87	0.040
Bacteria	Bacteroidetes	unknown	uncultured_Porphyromonadaceae_bacterium	-0.86	0.047
Bacteria	Firmicutes	Blautia	Blautia_obeum	-0.84	0.032
Bacteria	Actinobacteria	Mycobacterium	Mycobacterium_tuberculosis	1.46	0.043
Bacteria	Firmicutes	Massilimaliae	Massilimaliae_timonensis	1.59	0.030
Bacteria	Firmicutes	Intestinimonas	Intestinimonas_massiliensis	1.73	0.045
Bacteria	Firmicutes	Dorea	uncultured_Dorea_sp	1.77	0.034

Bacteria	Proteobacteria	Escherichia	Escherichia_coli	1.81	0.030
Bacteria	Firmicutes	Mediterraneibacter	Mediterraneibacter_butyricigenes	1.81	0.032
Bacteria	Firmicutes	Blautia	Blautia_hydrogenotrophica	1.81	0.032
Bacteria	Firmicutes	Lachnoclostridium	Clostridium_glycyrrhizinilyticum	1.84	0.022
Bacteria	Firmicutes	Flavonifractor	uncultured_Flavonifractor_sp	1.85	0.025
Bacteria	Firmicutes	Tyzzerella	Tyzzerella_nexilis	1.87	0.028
Bacteria	Firmicutes	Holdemania	Holdemania_filiformis	1.95	0.031
Bacteria	Firmicutes	unknown	Firmicutes_bacterium_AM31_12AC	1.95	0.032
Bacteria	Firmicutes	unknown	Eubacterium_siraeum	1.97	0.035
Bacteria	Firmicutes	unknown	Firmicutes_bacterium_CAG103	2.05	0.031
Bacteria	unknown	unknown	bacterium	2.08	0.026
Bacteria	Bacteroidetes	Barnesiella	Barnesiella_intestinihominis	2.21	0.025
Bacteria	Bacteroidetes	Alistipes	Alistipes_finegoldii	2.23	0.026
Bacteria	Actinobacteria	Collinsella	Collinsella_unknown_sp	2.35	0.021
Bacteria	Firmicutes	Anaerotignum	Anaerotignum_faecicola	2.42	0.024
Bacteria	Bacteroidetes	Alistipes	Alistipes_onderdonkii	3.05	<0.001
Bacteria	Firmicutes	Neglecta	Neglecta_timonensis	3.93	<0.001
Bacteria	Firmicutes	unknown	Firmicutes_bacterium_CAG83	4.09	<0.001
Bacteria	Firmicutes	Phascolarctobacterium	Phascolarctobacterium_succinatutens	4.21	<0.001
Bacteria	Firmicutes	Lachnotalea	Lachnotalea_unknown_sp	4.23	<0.001
Bacteria	Firmicutes	Coprococcus	Coprococcus_comes	4.28	<0.001
Bacteria	Firmicutes	Phascolarctobacterium	Phascolarctobacterium_faecium	4.72	<0.001
Bacteria	Firmicutes	Ruminococcus	Ruminococcus_torques_CAG61	5.13	<0.001

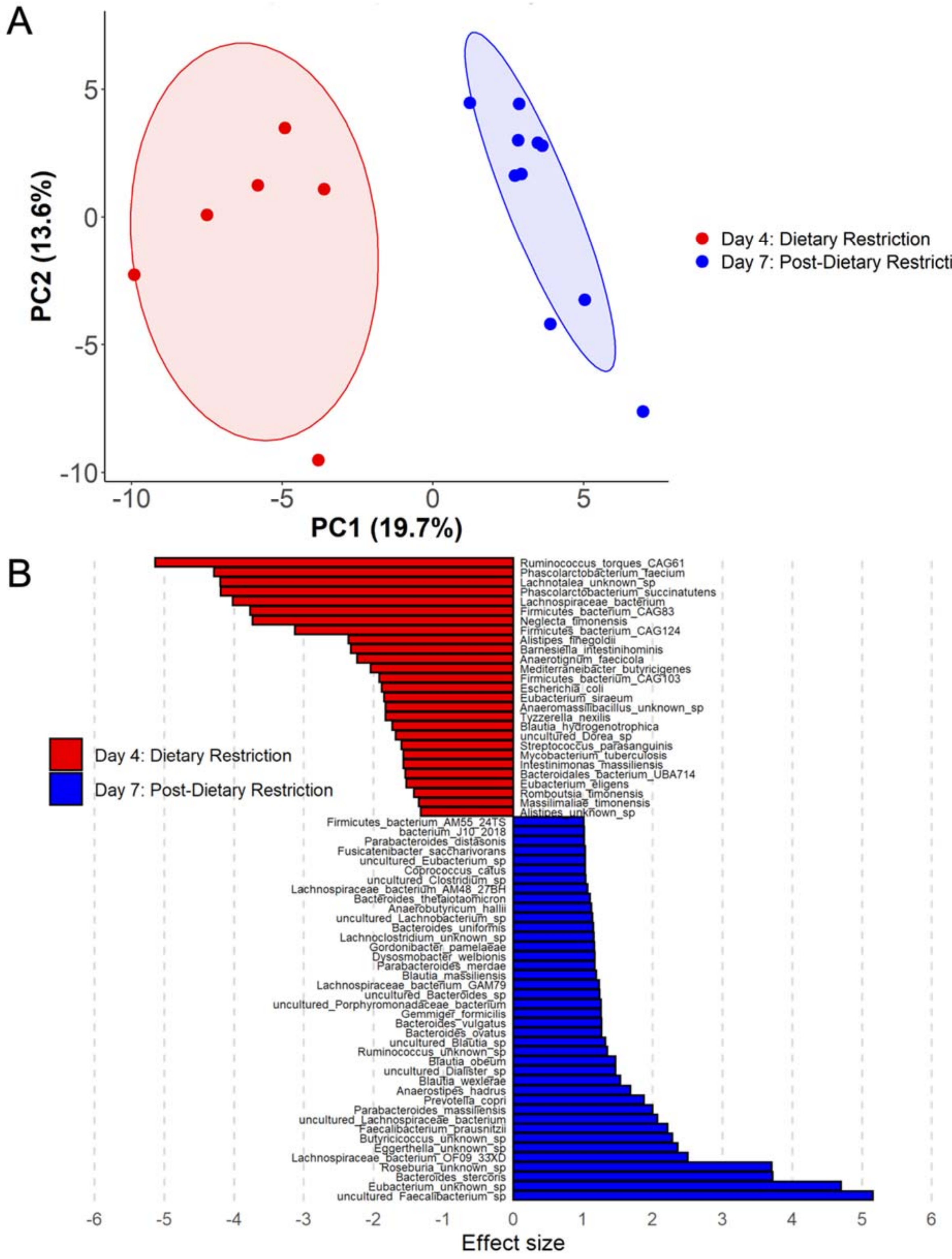

Supplemental Figure S4. Day-4 v. Day-7. Principal component analysis of species present between the two conditions (**A**). Effect size graph of significantly different species (FDR p<0.05, effect size >1) (**B**).

Supplemental Table S3. Significantly different species between Day-4 and Day-7 conditions (FDR p<0.05).

Kingdom	Phylum	Genus	Species	Effect Size	FDR p-value
Bacteria	Firmicutes	Ruminococcus	Ruminococcus_torques_CAG61	-5.13	<0.001
Bacteria	Firmicutes	Phascolarctobacterium	Phascolarctobacterium_faecium	-4.29	<0.001
Bacteria	Firmicutes	Lachnotalea	Lachnotalea_unknown_sp	-4.20	<0.001
Bacteria	Firmicutes	Phascolarctobacterium	Phascolarctobacterium_succinatutens	-4.19	<0.001
Bacteria	Firmicutes	unknown	Lachnospiraceae_bacterium	-4.02	<0.001
Bacteria	Firmicutes	unknown	Firmicutes_bacterium_CAG83	-3.77	<0.001
Bacteria	Firmicutes	Neglecta	Neglecta_timonensis	-3.74	<0.001
Bacteria	Firmicutes	unknown	Firmicutes_bacterium_CAG124	-3.13	<0.001
Bacteria	Bacteroidetes	Alistipes	Alistipes_finegoldii	-2.36	0.032
Bacteria	Bacteroidetes	Barnesiella	Barnesiella_intestinihominis	-2.32	0.024
Bacteria	Firmicutes	Anaerotignum	Anaerotignum_faecicola	-2.24	0.029
Bacteria	Firmicutes	Mediterraneibacter	Mediterraneibacter_butyricigenes	-2.04	0.038
Bacteria	Firmicutes	unknown	Firmicutes_bacterium_CAG103	-1.92	0.032
Bacteria	Proteobacteria	Escherichia	Escherichia_coli	-1.88	0.029
Bacteria	Firmicutes	unknown	Eubacterium_siraeum	-1.85	0.032
Bacteria	Firmicutes	Anaeromassilibacillus	Anaeromassilibacillus_unknown_sp	-1.83	0.035
Bacteria	Firmicutes	Tyzzerella	Tyzzerella_nexilis	-1.83	0.027
Bacteria	Firmicutes	Blautia	Blautia_hydrogenotrophica	-1.73	0.042
Bacteria	Firmicutes	Dorea	uncultured_Dorea_sp	-1.68	0.036
Bacteria	Firmicutes	Streptococcus	Streptococcus_parasanguinis	-1.60	0.041
Bacteria	Actinobacteria	Mycobacterium	Mycobacterium_tuberculosis	-1.58	0.032
Bacteria	Firmicutes	Intestinimonas	Intestinimonas_massiliensis	-1.57	0.044
Bacteria	Bacteroidetes	unknown	Bacteroidales_bacterium_UBA714	-1.54	0.047
Bacteria	Firmicutes	Eubacterium	Eubacterium_eligens	-1.53	0.034
Bacteria	Firmicutes	Romboutsia	Romboutsia_timonensis	-1.42	0.044
Bacteria	Firmicutes	Massilimaliae	Massilimaliae_timonensis	-1.35	0.037
Bacteria	Bacteroidetes	Alistipes	Alistipes_unknown_sp	-1.32	0.048
Bacteria	Actinobacteria	Eggerthella	Eggerthella_lenta	0.72	0.046
Bacteria	Firmicutes	Blautia	Blautia_unknown_sp	0.89	0.043
Bacteria	Firmicutes	unknown	Clostridiales_bacterium_VE202_03	0.98	0.022
Bacteria	unknown	unknown	bacterium_J10_2018	1.01	0.019
Bacteria	Bacteroidetes	Parabacteroides	Parabacteroides_distasonis	1.01	0.026
Bacteria	Firmicutes	Fusicatenibacter	Fusicatenibacter_saccharivorans	1.03	0.020
Bacteria	Firmicutes	Eubacterium	uncultured_Eubacterium_sp	1.03	0.029
Bacteria	Firmicutes	Coprococcus	Coprococcus_catus	1.03	0.023
Bacteria	Firmicutes	Clostridium	uncultured_Clostridium_sp	1.04	0.017
Bacteria	Bacteroidetes	Bacteroides	Bacteroides_thetaiotaomicron	1.10	0.013
Bacteria	Firmicutes	Anaerobutyricum	Anaerobutyricum_hallii	1.12	0.020

Bacteria	Firmicutes	Lachnobacterium	uncultured_Lachnobacterium_sp	1.14	0.013
Bacteria	Bacteroidetes	Bacteroides	Bacteroides_uniformis	1.15	0.018
Bacteria	Firmicutes	Dysosmobacter	Dysosmobacter_welbionis	1.17	0.011
Bacteria	Firmicutes	Blautia	Blautia_massiliensis	1.19	0.015
Bacteria	Bacteroidetes	Bacteroides	uncultured_Bacteroides_sp	1.24	0.010
Bacteria	Bacteroidetes	unknown	uncultured_Porphyromonadaceae_bacterium	1.26	0.011
Bacteria	Firmicutes	Gemmiger	Gemmiger_formicilis	1.26	0.009
Bacteria	Bacteroidetes	Bacteroides	Bacteroides_vulgatus	1.26	0.011
Bacteria	Bacteroidetes	Bacteroides	Bacteroides_ovatus	1.27	0.010
Bacteria	Firmicutes	Blautia	uncultured_Blautia_sp	1.32	0.009
Bacteria	Firmicutes	Ruminococcus	Ruminococcus_unknown_sp	1.35	0.008
Bacteria	Firmicutes	Blautia	Blautia_obeum	1.47	0.008
Bacteria	Firmicutes	Dialister	uncultured_Dialister_sp	1.47	0.005
Bacteria	Firmicutes	Blautia	Blautia_wexlerae	1.53	0.004
Bacteria	Firmicutes	Anaerostipes	Anaerostipes_hadrus	1.68	0.001
Bacteria	Bacteroidetes	Prevotella	Prevotella_copri	1.88	0.002
Bacteria	Bacteroidetes	Parabacteroides	Parabacteroides_massiliensis	2.00	0.002
Bacteria	Firmicutes	unknown	uncultured_Lachnospiraceae_bacterium	2.07	<0.001
Bacteria	Firmicutes	Faecalibacterium	Faecalibacterium_prausnitzii	2.21	0.001
Bacteria	Firmicutes	Butyricicoccus	Butyricicoccus_unknown_sp	2.28	0.001
Bacteria	Actinobacteria	Eggerthella	Eggerthella_unknown_sp	2.36	0.001
Bacteria	Firmicutes	unknown	Lachnospiraceae_bacterium_OF09_33XD	2.50	0.002
Bacteria	Firmicutes	Roseburia	Roseburia_unknown_sp	3.71	<0.001
Bacteria	Bacteroidetes	Bacteroides	Bacteroides_stercoris	3.72	<0.001
Bacteria	Firmicutes	Eubacterium	Eubacterium_unknown_sp	4.70	<0.001
Bacteria	Firmicutes	Faecalibacterium	uncultured_Faecalibacterium_sp	5.16	<0.001

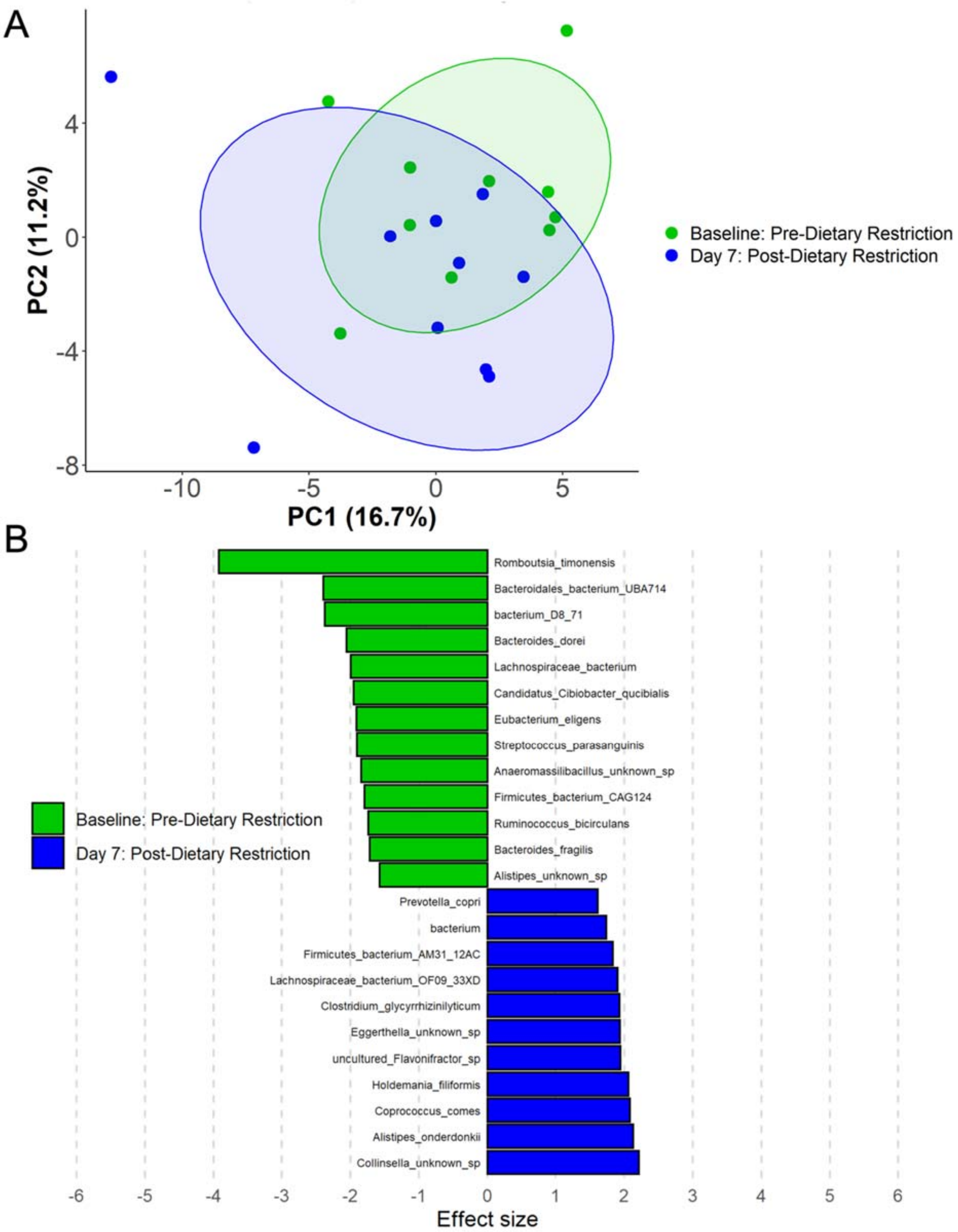

Supplemental Figure S5. Baseline v. Day-7. Principal component analysis of species present between the two conditions (**A**). Effect size graph of significantly different species (FDR p<0.05, effect size >1) (**B**).

Supplemental Table S4. Significantly different species between Baseline and Day-7 conditions (FDR p<0.05).

Kingdom	Phylum	Genus	Species	Effect Size	FDR p-value
Bacteria	Firmicutes	Romboutsia	Romboutsia_timonensis	-3.92	<0.001
Bacteria	Bacteroidetes	unknown	Bacteroidales_bacterium_UBA714	-2.39	0.002
Bacteria	unknown	unknown	bacterium_D8_71	-2.38	<0.001
Bacteria	Bacteroidetes	Bacteroides	Bacteroides_dorei	-2.05	0.002
Bacteria	Firmicutes	unknown	Lachnospiraceae_bacterium	-1.99	0.001
Bacteria	Firmicutes	Candidatus Cibiobacter	Candidatus_Cibiobacter_qucibialis	-1.95	0.003
Bacteria	Firmicutes	Eubacterium	Eubacterium_eligens	-1.91	0.004
Bacteria	Firmicutes	Streptococcus	Streptococcus_parasanguinis	-1.91	0.005
Bacteria	Firmicutes	Anaeromassilibacillus	Anaeromassilibacillus_unknown_sp	-1.85	0.004
Bacteria	Firmicutes	unknown	Firmicutes_bacterium_CAG124	-1.79	0.005
Bacteria	Firmicutes	Ruminococcus	Ruminococcus_bicirculans	-1.74	0.008
Bacteria	Bacteroidetes	Bacteroides	Bacteroides_fragilis	-1.72	0.005
Bacteria	Bacteroidetes	Alistipes	Alistipes_unknown_sp	-1.57	0.008
Bacteria	Bacteroidetes	Prevotella	Prevotella_copri	1.61	0.004
Bacteria	unknown	unknown	bacterium	1.73	0.004
Bacteria	Firmicutes	unknown	Firmicutes_bacterium_AM31_12AC	1.83	0.004
Bacteria	Firmicutes	unknown	Lachnospiraceae_bacterium_OF09_33XD	1.90	0.001
Bacteria	Firmicutes	Lachnoclostridium	Clostridium_glycyrrhizinilyticum	1.93	0.002
Bacteria	Actinobacteria	Eggerthella	Eggerthella_unknown_sp	1.94	0.002
Bacteria	Firmicutes	Flavonifractor	uncultured_Flavonifractor_sp	1.94	0.001
Bacteria	Firmicutes	Holdemania	Holdemania_filiformis	2.06	0.002
Bacteria	Firmicutes	Coprococcus	Coprococcus_comes	2.08	0.002
Bacteria	Bacteroidetes	Alistipes	Alistipes_onderdonkii	2.13	0.002
Bacteria	Actinobacteria	Collinsella	Collinsella_unknown_sp	2.21	0.001
